# Endometriosis– “Either way a tragedy”? A qualitative social media analysis of endometriosis perceptions in Germany

**DOI:** 10.1186/s12905-025-03865-2

**Published:** 2025-07-04

**Authors:** Till Neugebauer, Valeria Schellenberg, Alena Allak, Siegrun Pardon, Yüce Yilmaz-Aslan, Sven Schiermeier, Claudia Kiessling, Sven Schmiedl, Christoph Dockweiler, Patrick Brzoska

**Affiliations:** 1 Faculty of Health, School of Medicine, Health Services Research, Witten/Herdecke University, Witten, Germany; 2 Faculty of Health, School of Medicine, Gynaecology and Obstetrics, Witten/Herdecke University, Witten, Germany; 3 Department of Gynaecology and Obstetrics, Marien-Hospital Witten, Witten, Germany; 4 Faculty of Health, School of Medicine, Education of Personal and Interpersonal Competencies in Health Care, Witten/Herdecke University, Witten, Germany; 5 Faculty of Health, School of Medicine, Clinical Pharmacology & Center for Clinical Trials, Witten/Herdecke University, Witten, Germany; 6 Philipp Klee-Institute of Clinical Pharmacology, Helios University Hospital Wuppertal, Wuppertal, Germany; 7 Department of Social Sciences, Faculty of Arts and Humanities, University of Siegen, Siegen, Germany

**Keywords:** Endometriosis, Content analysis, Stigmatisation, Communication, Social media, Barriers

## Abstract

**Background:**

Endometriosis is one of the most common diseases in women of reproductive age. Those affected suffer from a variety of symptoms that can have a challenging impact on different areas of life. Social media such as Instagram, TikTok and YouTube offer affected women a platform for sharing their condition and increasing its visibility. This study examines how endometriosis is communicated in the comment sections of such posts on social media in Germany in order to better understand the reality of the lives of those affected.

**Methods:**

300 comments from Instagram, TikTok and YouTube were evaluated using summarizing qualitative content analysis. Data collection took place between October and November 2024.

**Results:**

Four categories were identified that shaped the exchange on social media. These included psychological and physical stress, criticism of healthcare, social impact and coping with endometriosis.

**Conclusions:**

Endometriosis remains stigmatised as a “women’s disease”, which makes recognition and prioritization in the healthcare system difficult. The reduction to menstruation-related complaints hinders the treatment of endometriosis-related symptoms, while pejorative communication by doctors increases the burden on those affected. While social media enables open dialogue and peer support, it also has potential to spread misinformation, influencing health decisions and fostering scepticism toward medical advice. Addressing these challenges requires a multifaceted public health response: destigmatising endometriosis through education, improving medical training, strengthening diagnostic pathways, and raising awareness in society.

## Background

Endometriosis is one of the most common chronic diseases among women of reproductive age. Worldwide, approximately 10–15% of women are affected [1]. In Germany, more than 53,000 new cases were diagnosed in 2022, with a high number of unreported cases suspected [2]. The growth of endometrium-like tissue outside the uterus can trigger inflammation, leading to cycle-independent symptoms such as painful menstruation, chronic lower abdominal pain, pain during intercourse, discomfort during bowel movements or urination, and fertility issues [3]. Despite its prevalence and the significant suffering of those affected, endometriosis often remains undiagnosed. On average, it takes 7–10 years to receive a diagnosis [3]. The reasons for this are multifactorial and not always clearly identifiable. From a medical perspective, misdiagnoses due to insufficient knowledge about endometriosis and a lack of awareness among healthcare professionals contribute to diagnostic delays [4]. This is further complicated by the fact that imaging techniques such as ultrasound or MRI may not reliably detect endometrial lesions and interpreting them requires specialised knowledge [1]. In Germany, endometriosis care is embedded in its statutory health insurance system, with general gynaecologists or general practitioners serving as primary points of contact. While annual preventive check-ups are covered, they often do not systematically address symptoms of endometriosis [5]. Specialised endometriosis centres exist but are unevenly distributed and difficult to access without a referral. As a result, early detection and consistent treatment remain challenging despite formal access to care.

Societally, the taboo surrounding menstrual pain and the stigmatisation of gynaecological complaints hinder the recognition and discussion of the disease [4]. Such stigmatisation has ethical, societal, and clinical implications: it affects not only the timing and quality of diagnosis but also the dignity and autonomy of those affected, leading to inadequate care. The concept of stigma is therefore central to understanding the structural and interpersonal barriers faced by those affected. Drawing on Goffman’s definition, stigma constitutes a “profoundly discrediting” attribute that leads to the devaluation of individuals in specific social contexts [6]. In the case of endometriosis, stigma is frequently associated with gendered assumptions about pain tolerance and emotional expression. As the condition predominantly affects women, its symptoms are often trivialized or dismissed as exaggerated, normal, or psychosomatic [7, 8].

In addition to physical symptoms, endometriosis also imposes psychosocial burdens on those affected. The loss of quality of life, social isolation, misunderstandings in personal relationships, and financial constraints due to inadequate treatment options are common consequences [9]. To date, little is known about the lived experiences of affected individuals in Germany [10–12]. However, these perceptions are crucial to understand the needs of those affected and to improve health care services accordingly. Valuable insights could be gained from social media platforms such as Instagram and YouTube, which are increasingly used by affected women to seek information and support regarding endometriosis [13].

## Methods

### Aim

The aim of this study was to explore the perception and discussion of endometriosis based on social media posts. The study sought to identify which aspects are highlighted by affected women, as well as by other individuals with or without direct experience of the condition, in the comment sections of these networks. Additionally, it aimed to explore the challenges faced in healthcare provision and societal attitudes towards endometriosis. This research could contribute to a better understanding of the experiences of those affected, the barriers to care, and the societal stigmatization of endometriosis.

### Design and data selection

This study used a qualitative research design with a focus on content from social media. The comment sections of three social media platforms (Instagram, YouTube, and TikTok) were examined regarding users’ experiences and perceptions of healthcare provision and coping with endometriosis. These social networks were chosen as they are one of the most used platforms among women of reproductive age in Germany [14] and also worldwide [13, 15]. To identify content related to endometriosis, German equivalents of keywords and hashtags such as “endometriosis,” “endometriosis care,” “endometriosis experience,” and “endo” were used. Only German-language posts were considered in data collection to explore experiences within Germany’s gynaecological healthcare structures.

On Instagram, in addition to keyword searches, channels identified by a German mobile app called Endo-App as the “Who is Who” of endometriosis influencers in Germany were also examined [16]. The Endo-App is a certified Digital Health Application (DiGA) in Germany, which must be prescribed by a physician and is reimbursable by statutory health insurance. It supports patients in managing their endometriosis by providing educational content, symptom tracking tools, exercise modules, and psychological guidance. The curated social media channels recommended within the app are therefore presumed to play a significant role in disseminating endometriosis-related content by the authors and are likely followed by affected individuals. On YouTube, posts were sorted using the platform’s relevance algorithm, which ranks content based on keyword matching in titles, descriptions, and content, as well as interaction frequency.

**Fig. 1:**
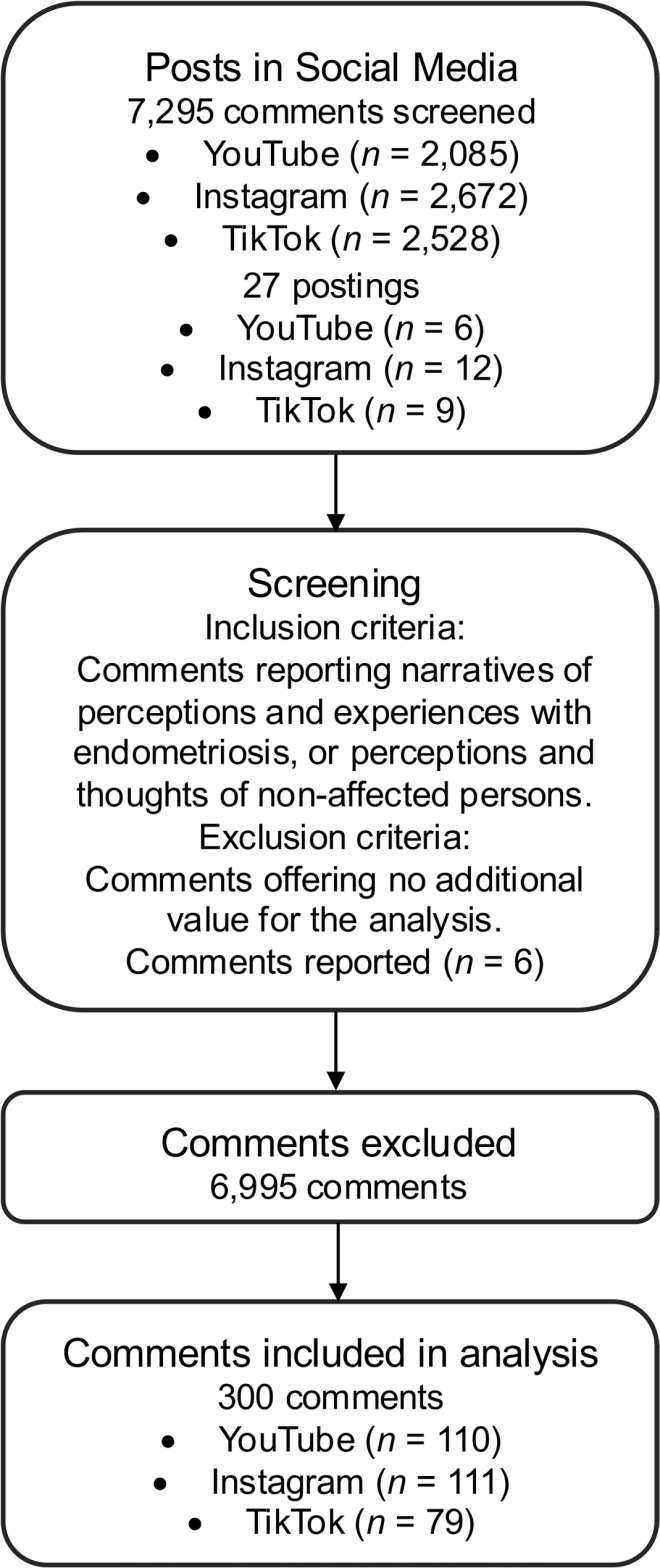
Flow diagram illustrating the selection procedure

Data collection took place between October 24 and November 18, 2024, covering content posted between 2019–2024. All comments relevant to the research objective that appeared in the comment sections of the identified posts on YouTube, Instagram and TikTok were reviewed and extracted for analysis. The posts themselves, which consist of videos and pictures, were not included in the analysis. In total, 7,295 comments from 27 postings were screened (Fig. 1). Comments were included in which narratives of perceptions and experiences with endometriosis were reported, but also those in which non-affected persons communicated their perceptions and thoughts. Comments that offered no added value for the analysis were excluded. This included comments consisting of just a few words. Hate messages or derogatory statements towards the stories of those affected were not collected, as these were directed against the individual and not against care in general. These comments were reported to the platforms by the researchers. The search continued until data saturation was reached, meaning no new relevant aspects were identified. No restrictions were placed on the publication dates of posts and comments to ensure that the growing societal and media attention towards endometriosis in recent years was adequately reflected in the dataset.

### Data analysis

A total of 300 commented user narratives were included in the analysis: 79 from TikTok, 111 from Instagram, and 110 from YouTube. The majority of these comments was found on German speaking, public broadcasting channels and “medfluencer” accounts that focus on medical topics in content creation. On average, videos and pictures from these channels received 144,000 views on YouTube (max.: 538,000; min.: 12,000), 276,000 views on Instagram (max.: 1.3 million; min.: 5,000), and 831,000 views on TikTok (max.: 3 million; min.: 37,000). The analysis was conducted using the summarizing qualitative content analysis approach by Kuckartz and Rädiker [17]. Initially, a subset of extracted comments was inductively coded by two authors (TN, VS), who are both public health specialists and working as researchers at their assigned institution. In this function they gained extensive knowledge on qualitative analysis of social media based content. The coded text passages were then categorized into main and subcategories through a reflexive process, forming the coding framework used to analyse the entire data material. Data analysis was performed using the qualitative data analysis software Casquada. This process was carried out in the original language of the comments. The selected citations were translated into English by using a Large Language Model translator (Deepl.com) to present them in the results. All translations were cross-checked by the research team to ensure accurate translation of the content.

### Ethical considerations

Only publicly accessible posts and comments classified as open data under research ethics guidelines were used [18]. To protect user anonymity, all comments were anonymized, and usernames were replaced with a standardized identifier (“User #X”). As the comments did not allow for a clear gender assignment, gender-neutral language was used to include individuals with endometriosis who do not identify as female. Direct quotes were also adjusted to prevent identity tracking through search engines; however, these adjustments only involved punctuation and sentence structure, not content.

## Results

The analysis of experiences and reactions in the comment sections of endometriosis-related posts can be categorized into four key categories: psychological and physical strains, criticism of healthcare provision, social impacts, and coping strategies.

### Psychological and physical strains

Users described profound effects on their mental health. Their reactions primarily expressed despair and anger, triggered by society’s and the healthcare system’s indifference.

*“I am expected to simply endure the pain– as if that were normal. But it isn’t. It is not okay that women must wait years for answers. It is not okay for their complaints to be dismissed. This must change. You live in constant fear of your own body.” (User #42)*.

This “fear of one’s own body” primarily relates to the chronic pain associated with endometriosis. The feeling of fear and helplessness during, but also between, menstrual periods led to constant tension among those affected. This persistent burden also created the impression of not being taken seriously. As a result, several users reported experiencing psychological distress, including resignation, hopelessness, and mental health conditions such as depression, self-doubt, and symptoms resembling post-traumatic stress disorder. Some users described reaching a psychological breaking point, with a few explicitly mentioning suicidal tendencies.

*“At 38*,* I couldn’t take it anymore and had suicidal thoughts. The pain had worn me down so much that I wanted to end it all*,* including myself. Then I changed gynaecologists and finally got my diagnosis.” (User #65)*.

The physical effects also played a central role for users. For example, severe endometriosis could have caused organ damage, leading to serious health complications.

*“My entire abdominal cavity was affected by endometriosis lesions*,* to the point that part of my intestine had to be removed. A surgical incision was necessary to treat all affected areas.” (User #18)*.

In addition to these extreme cases, users described experiencing severe pain due to endometriosis, which had far-reaching consequences for their physical health. Chronic pain, for example as a result of adhesion in the fallopian tubes, was frequently mentioned. These persistent physical symptoms often triggered a cascade of further health issues and limitations. However, the link between chronic physical pain and mental health was especially pronounced when patients felt their suffering was not taken seriously by medical professionals. This lack of validation could intensify psychological distress and feelings of isolation. One user described such an experience:

*“Today I heard something really great again when I went to a gynaecologist with severe pain due to adhesions in my fallopian tube: ‘Just go home*,* sit down*,* have a cup of tea and it will be fine.’” (User #180)*.

These physical strains therefore were closely linked to the mental health of those affected. Users frequently criticized the low success rates of conventional treatments, particularly the reliance on hormonal medications.

*“I find it so shocking that the only solution doctors often suggest is: ‘Take the [birth control] pill*,* and it will get better.’ That can’t be all! When will there finally be real solutions for us?” (User #113)*.

The limited effectiveness of treatments led to additional pressure, which is reflected in the emotional expressions of users. In some cases, illnesses associated with endometriosis such as Hashimoto’s disease or cardiovascular conditions arose.

### Criticism of healthcare

Users criticized the healthcare system and care structures, particularly the limited access to specialized endometriosis centres and the lack of communication and support from general gynaecologists. They responded with disappointment and anger to the limited financial and scientific resources dedicated to improving care and recognizing the disease. The prolonged diagnostic process, which often took more than 10 years for most of the affected individuals, was described as particularly distressing. Many users attributed this delay not only to clinical gaps but also to broader societal dynamics. For example, the tendency to minimise or dismiss conditions that predominantly affect women.

*“If this was a men’s disease*,* there would likely be plenty of research funding and numerous treatment options– but as a woman*,* you remain a second-class citizen.” (User #5)*.

This quote reflects a frequently expressed perception among users: that gender bias in medical research and healthcare provision contributes to the systemic neglect of endometriosis. The condition, as a so-called “women’s disease”, is seen as under prioritised within both scientific and clinical domains. This sentiment links directly to the stigmatisation of menstruation and chronic pelvic pain, which are still commonly trivialised in clinical encounters as perceived by the users. Some users even reported experiencing psychological manipulation in the form of medical gaslighting as their complaints were dismissed, minimised, or reframed as purely psychological.

*“I have suffered from endometriosis for 25 years*,* and even though my diagnosis has long been confirmed*,* I continue to experience severe medical gaslighting. My other chronic illnesses*,* such as irritable bowel syndrome*,* histamine intolerance*,* silent reflux*,* and chronic pain*,* are neither taken seriously nor adequately treated.” (User #203)*.

Many affected individuals who sought gynaecological care for their symptoms believed that this contributes to doctors’ general lack of interest. They argued that many doctors lack adequate knowledge about the disease, its diagnosis, and available treatment options. On the one hand this can result in uncertainty regarding endometriosis on the part of medical professionals, potentially manifesting in dismissive communication. On the other hand, doctors could not see the need for further examinations or referral to specialized endometriosis clinics, which could shift the responsibility for maintaining care onto patients. However, such behaviour imposed additional psychological strain on those affected.

*“After nine years of pain and constant claims from doctors that my complaints were psychological*,* a laparoscopy finally confirmed endometriosis. I’m afraid to hand my doctor the letter with the diagnosis because he has always dismissed my symptoms. Either way*,* endometriosis and everything surrounding it is just one big tragedy.” (User #26)*.

### Social impacts

The daily lives of affected women were significantly restricted by endometriosis. Users reported that leisure activities or everyday tasks were hardly manageable due to the pain. As a result, those affected often relied on external help from partners or family members, which had a negative impact on their interpersonal relationships.

*“I remember collapsing from the worst pain of my life*,* and my boyfriend nearly had a heart attack because he was terrified I would die on him.” (User #292)*.

Consequently, many withdrew from social interactions, which was further reinforced by a lack of understanding from their surroundings. This also extended to the workplace. Frequent absences led to fears of losing one’s job. Users criticized both the lack of employer awareness and the absence of measures to accommodate these absences.

Another aspect criticized by users is the societal expectation that women of reproductive age should desire children. Some affected individuals reported being told by doctors that pregnancy could serve as a treatment for endometriosis.

*“At 24*,* I was admitted to the women’s clinic as an emergency case. During the discharge conversation*,* the doctor told me: ‘If you want to get better*,* you should get pregnant.’” (User #180)*.

However, often the woman’s age or whether motherhood aligns with her personal life choices was not considered. Women without a desire to have children faced societal expectations of motherhood, frequently mentioned in the comments, which can add further mental pressure. Meanwhile, women who do wish to have children experienced additional stress when infertility or difficulties conceiving due to endometriosis come into play.

### Coping with endometriosis

Despite these challenges, many affected individuals actively sought alternative ways to improve their quality of life. The exchange of self-help tips, such as dietary changes, herbal remedies, or yoga exercises, played a central role in coping with endometriosis. Many users also highlighted the importance of endometriosis support groups. Overall, the comments reflected a strong sense of awareness, mutual support, and gratitude for shared advice and experiences.

*“My gynaecologist created a supplement plan for me*,* and I strictly follow an anti-inflammatory diet. Various birth control pills had no effect on me. I have to give up a lot*,* but physically*,* this approach has helped me a lot. I wish you all the best*,* no matter which path you choose!” (User #269)*.

Nevertheless, user narratives also revealed that the search for effective treatments often came with financial burdens and emotional strain. The complexity of the disease and the difficulty in accessing specialized care were repeatedly described as key barriers to finding an individually suitable treatment. Many users emphasised how exhausting it can be to try multiple approaches over time, particularly when these fail to provide relief or meaningful results.

## Discussion

This study highlights the complex interactions between the burdens of endometriosis, perceptions of the disease, and the ways society and the healthcare system handle it. A key finding is that the perceptions of the users reveal an ongoing stigmatisation of endometriosis as a condition primarily affecting women. This stigma not only hinders the recognition and prioritisation of the disease within the healthcare system [19] but can also negatively impact the mental health of those affected, particularly when they experience discrimination due to their condition [20]. The reported average diagnosis time of up to 10 years reflects this issue and aligns with current research findings [3, 20]. Although the term stigma was not explicitly used by the users, their identification as women and the associated social prejudices can be understood as stigmatising experiences. This was evident in their reports of having their symptoms dismissed by healthcare providers. Stigmatisation occurred when individuals disclosed reproductive health problems that society often views negatively [11], such as the normalisation of menstrual pain [7] or the perception that women complain excessively about issues traditionally associated with their gender [8]. Although the consideration of endometriosis in diagnostics for Germany, Austria and Switzerland has been laid down in an S2K guideline of the relevant specialist societies [21], societal attitudes toward conditions affecting women can still influence perceptions, both among patients and healthcare providers. The affected individuals in this study emphasized this deficit, criticizing their interactions with healthcare providers, which they perceived as dismissive and invalidating. This issue has been widely reported in international endometriosis care emphasizing the importance of raising awareness of the needs for sensitive and empathic interactions among healthcare professionals [22]. In Germany, however, it remains unclear how patient-provider communication impacts stigma and associated burdens [23].

To address these challenges, the reduction of endometriosis related stigma seems essential, recognising it as a chronic and holistic disease rather than merely a menstrual issue [8]. Some progress is evident on social media, where discussions about endometriosis are becoming more open and less shame associated [24]. Initiatives and campaigns such as the Danish Endometriosis Patients Association’s #1in10 campaign also demonstrate the increased presence on social media and aims to contribute to the destigmatisation of the condition [25]. The experiences explored in this analysis further support this trend. Nevertheless, the quality of the information that young women can access online should be taken into account. There is a risk that false information can be shared via posts and comments on social media [26, 27], which could potentially influence gynaecological care utilization behaviour or enhance scepticism towards hormonal treatments [28]. The extent to which information about endometriosis on social media in Germany is reliable still needs to be investigated further, especially with regard to current developments in the quality assurance of information on the internet [29].

The evaluated comments reveal a persistent high level of distress due to psychological, emotional, and social burdens, leading to a significant reduction in quality of life [6]. The everyday challenges described align with known barriers in the literature [4, 10]. However, current healthcare structures in Germany do not seem to meet the needs of those affected. While access to specialised endometriosis centres in Germany, Austria and Switzerland is improving due to the continuous certification of new facilities, they remain difficult to reach for many patients. One possible reason could be the perception that referrals are limited due to a lack of interest or engagement from general gynaecologists as stated by the users in this study. Another contributing aspect could be limited awareness of endometriosis among those affected. This lack of awareness may prevent patients from accurately describing their symptoms, leading to missed indications for endometriosis-related investigations [3]. Given the existing challenges of limited time resources and financial pressures from health insurance providers, raising awareness among patients and enhancing the diagnostic skills of gynaecologists are essential measures to ensure that relevant symptoms are recognised and appropriately investigated [4]. In this context, Hudelist et al. [3] recommend public awareness initiatives to help reduce barriers to accessing endometriosis care. A stronger focus on multi- or interdisciplinary collaboration between doctors and nurses specializing in endometriosis, as is already being implemented in the UK and demanded by the S2K guideline to certify as an endometriosis centre in Germany, could also have a beneficial effect [30]. Given the prevalence of endometriosis among women, such cooperation could be a meaningful addition to gynaecological care, improving treatment quality, reducing diagnosis times, enhancing communication, and ensuring more comprehensive and patient-centred care. As challenges appear to arise primarily in the processes between primary and specialist care, multi- and interdisciplinary approaches could also be pursued outside of endometriosis centres. This would also offer the opportunity to share the extensive knowledge from the endometriosis centres with the surrounding care structures and to initiate treatment at an early stage [31]. Positive examples of such approaches already exist in the management of other chronic conditions [32]. This discussion aligns with the ongoing demands for advanced nursing practices in Germany and the shift toward comprehensive care, emphasizing collaborative decision-making between physicians and other healthcare professionals [33]. Further research should also be conducted into how patients can be given easier access to specialised care, e.g. by increasing their own awareness or their ability to recognise symptoms, seek out reliable information, and make informed health decisions [34]. Independent services for better information about endometriosis, such as the website *informedhealth.org* of the Institute for Quality and Efficiency in Health Care (IQWiG, Germany), are already trying to create more awareness for endometriosis. However, it remains unclear through which channels this information can be communicated to the affected group and what impact this has on the utilisation of care services.

A limitation of this study is the lack of assessment regarding the quality and intent of the comments, which may also be influenced by users’ distress. Although social media provides valuable insights into the perceptions and experiences of endometriosis patients and non-affected individuals, underscoring the demand for increased visibility, this could have led to an overestimation of the results. In addition, the content of the videos and postings themselves was not assessed, which is why no statements can be made about the accuracy of the information disseminated there. It is therefore difficult to understand to what extent the comments and reactions were influenced by the content. Particularly in the case of posts on medical influencer channels, it must be noted that these channels sometimes address a specific audience that often already has an existing opinion on a topic and spreads it in the comments [35]. Moreover, these channels often do not undergo any quality or fact checks, which is why there can be no assurance that their content is correct. Comments can also be written by so-called “trolls” who deliberately take a provocative stance, especially on sensitive topics. It was noticeable in the comments that hate messages and insults were primarily written by supposedly male users. The reactions to these comments could have led to further stress reactions among the users, resulting in further overestimation of the results. It is also essential to note that certain groups such as individuals who refrain from public engagement due to stigma, digital exclusion, or personal reasons remain underrepresented in the data. This underrepresentation may contribute to an underestimation of the broader burden of the disease. Further research should consider triangulating social media data with other qualitative or clinical sources to contextualise these perceptions within broader healthcare structures and to assess the reliability and representativeness of online narratives more comprehensively. Finally, it should be noted that the platforms work with algorithms that highlight or suppress posts based on various factors such as views, length and popularity. This can lead to a distortion in the posts displayed.

## Conclusion

This study highlights the persistent stigma surrounding endometriosis, contributing to delayed diagnosis, inadequate healthcare prioritisation, and dismissive provider interactions in Germany. While social media fosters open discussions and can help reduce stigma, misinformation remains a concern, potentially influencing healthcare decisions and causing treatment scepticism. The significant emotional and social burdens of endometriosis underscore the need for more patient-centred care. Although specialised endometriosis centres are becoming increasingly available, challenges persist in the referral process between general gynaecologists and these centres, limiting timely access to advanced care. Strengthening both patients’ awareness and health literacy regarding endometriosis is crucial, as better knowledge can help individuals recognise and articulate their symptoms more accurately, facilitating earlier diagnosis and appropriate referrals. Enhancing the diagnostic expertise of general gynaecologists and promoting interdisciplinary collaboration are also essential for improving diagnosis and treatment outcomes. Given the influence of social media, further research is needed to assess the reliability of online health information and its impact on patient perceptions.

In conclusion, increasing education and awareness of endometriosis among patients and their relatives, healthcare professionals and in politics is vital. By empowering patients to communicate their symptoms more effectively and improving diagnostic pathways, healthcare systems can deliver more timely and targeted care. Additionally, leveraging insights from social media can help shape sustainable healthcare solutions that better address patient needs.

## Data Availability

The data material used in the form of comments is publicly accessible on the respective social media platforms. This data relates to November 18.
